# Supplementary material for: Longevity of different in-office treatments for dentin hypersensitivity: A 6-month randomized and parallel clinical trial
Source: PLoS One. 2026 Feb 17;21(2):e0342651. doi: 10.1371/journal.pone.0342651 (PMC12912554; doi:10.1371/journal.pone.0342651)
Supplement: S1 File — Original ethics committee approval document in Portuguese. (PDF) [file pone.0342651.s001.pdf]

PARECER CONSUBSTANCIADO DO CEP

DADOS DO PROJETO DE PESQUISA

Título da Pesquisa: Efeito de diferentes protocolos no tratamento da hipersensibilidade dentinária: avaliação in situ e in vivo

Pesquisador: FERNANDA DE SOUZA E SILVA RAMOS

Área Temática:

Versão: 1

CAAE: 30122220.1.0000.5420

Instituição Proponente:

Patrocinador Principal: Financiamento Próprio

DADOS DO PARECER

Número do Parecer: 3.988.387

Apresentação do Projeto:

Objetivo: Investigar os efeitos da aplicação de diferentes agentes dessensibilizantes sobre exposições radiculares hipersensíveis. Métodos: Para o estudo in situ, 72 espécimes de dentina bovina com túbulos dentinários abertos serão subdivididos de acordo com os seguintes grupos: verniz fluoretado (Duraphat, Colgate, Colgate-Palmolive Company); verniz fluoretado 5% de NaF e 5% de trimetafosfato de sódio nanoparticulado (TMP) e um verniz fotopolimerizável (PRG Barrier Coat, Shofu INC.). Após a análise inicial de microdureza para seleção dos blocos, os espécimes receberão uma camada de verniz ácido resistente e serão submetidos a ciclos erosivo/abrasivo. Ao final dos ciclos, os espécimes serão analisados por meio da perfilometria e dureza interna. O grau de cooperação do voluntário será avaliado através do microsensor TheraMon®. Para o estudo in vivo, 45 pacientes com pelo menos dois dentes com dentina radicular exposta não cavitadas e com hipersensibilidade dentinária serão selecionados para este estudo, totalizando 90 dentes, os quais serão divididos de acordo com os grupos do estudo in situ. As aplicações serão realizadas e avaliadas nos seguintes tempos: inicial, após 7, 15, 30 dias e 6 meses. As análises qualitativas serão realizadas por meio da escala visual analógica e computadorizada (VAS e CoVAS). A análise quantitativa será realizada por meio de um equipamento de análise neurosensorial. Após coleta dos dados, os resultados serão submetidos aos testes estatísticos adequados para cada tipo de

análise. Relevância clínica: O presente estudo possui potencial de obtenção de protocolo clínico inovador e eficaz para o tratamento da hipersensibilidade dentinária.

Objetivo da Pesquisa:

Objetivo Primário:

O objetivo do estudo in situ será avaliar a perfilometria e a dureza interna da dentina após a aplicação de três produtos com potencial dessensibilizante (Verniz de Flúor Duraphat, Verniz de 5% NaF com 5% de TMP nanoparticulado, PRG Barrier Coat) submetidos ao desafio erosivo/abrasivo. O objetivo do estudo in vivo será quantificar a sensibilidade dentinária após aplicação de três produtos com potencial dessensibilizante (Verniz de Flúor Duraphat, Verniz de 5% NaF com 5% de TMP nanoparticulado, PRG Barrier Coat) utilizando a Escala visual analógica e computadorizada (VAS e CoVAS), e um equipamento de análise neurossensorial.

Avaliação dos Riscos e Benefícios:

Riscos:

Riscos mínimos, que são próprios de qualquer tratamento odontológico de rotina

Benefícios:

Os pacientes terão os dentes tratados quanto a sensibilidade. Faremos também o encaminhamento para as outras disciplinas, caso necessite de outro tipo de tratamento odontológico.

Comentários e Considerações sobre a Pesquisa:

Pesquisa apresenta-se apta para a sua realização.

Considerações sobre os Termos de apresentação obrigatória:

Todos os termos foram adicionados de acordo com a resolução 466/12 do Conselho Nacional de Saúde.(CNS).

Recomendações:

Não há.

Conclusões ou Pendências e Lista de Inadequações:

Pesquisa apresenta-se apta para a sua realização.

Considerações Finais a critério do CEP:

Salientamos que, de acordo com a Resolução 466 CNS, de 12/12/2012 (título X, seção X.1., art. 3, item b, e, título XI, seção XI.2., item d), há necessidade de apresentação de relatórios semestrais, devendo o primeiro relatório ser enviado até 01/10/2020.

Endereço: JOSE BONIFACIO 1193
Bairro: VILA MENDONCA **CEP:** 16.015-050
UF: SP **Município:** ARACATUBA
Telefone: (18)3636-3200 **Fax:** (18)3636-3332 **E-mail:** andrebertoz@foa.unesp.br

Este parecer foi elaborado baseado nos documentos abaixo relacionados:

Tipo Documento	Arquivo	Postagem	Autor	Situação
Informações Básicas do Projeto	PB_INFORMAÇÕES_BÁSICAS_DO_PROJETO_1529447.pdf	20/03/2020 11:07:21		Aceito
TCLE / Termos de Assentimento / Justificativa de Ausência	TCLE.pdf	20/03/2020 10:48:42	FERNANDA DE SOUZA E SILVA RAMOS	Aceito
Projeto Detalhado / Brochura Investigador	ProjetodePesquisa.pdf	20/03/2020 10:48:24	FERNANDA DE SOUZA E SILVA RAMOS	Aceito
Folha de Rosto	FolhaRosto.pdf	20/03/2020 10:46:30	FERNANDA DE SOUZA E SILVA RAMOS	Aceito

Situação do Parecer:

Aprovado

Necessita Apreciação da CONEP:

Não

ARACATUBA, 24 de Abril de 2020

Assinado por:
Aldiéris Alves Pesqueira
(Coordenador(a))
