# Supplementary material for: Longevity of different in-office treatments for dentin hypersensitivity: A 6-month randomized and parallel clinical trial
Source: PLoS One. 2026 Feb 17;21(2):e0342651. doi: 10.1371/journal.pone.0342651 (PMC12912554; doi:10.1371/journal.pone.0342651)
Supplement: S2 File — Original final report submitted to the ethics committee in Portuguese. (PDF) [file pone.0342651.s002.pdf]

PARECER CONSUBSTANCIADO DO CEP

DADOS DO PROJETO DE PESQUISA

Título da Pesquisa: Efeito de diferentes tratamentos na hipersensibilidade dentinária: avaliação da citotoxidade e estudo clínico randomizado

Pesquisador: FERNANDA DE SOUZA E SILVA RAMOS

Área Temática:

Versão: 4

CAAE: 30122220.1.0000.5420

Instituição Proponente: I

Patrocinador Principal: Financiamento Próprio

DADOS DA NOTIFICAÇÃO

Tipo de Notificação: Envio de Relatório Final

Detalhe:

Justificativa: Encaminho em anexo o relatório final do projeto intitulado "Efeito de diferentes

Data do Envio: 10/08/2023

Situação da Notificação: Parecer Consubstanciado Emitido

DADOS DO PARECER

Número do Parecer: 6.267.262

Apresentação da Notificação:

Apresentação do Relatório final das atividades de pesquisa: Efeito de diferentes tratamentos na hipersensibilidade dentinária: avaliação da citotoxidade e estudo clínico randomizado.

Objetivo da Notificação:

Relatório Final

Avaliação dos Riscos e Benefícios:

Risco Mínimo.

Comentários e Considerações sobre a Notificação:

Relatório Final aprovado.

Endereço: JOSE BONIFACIO 1193
Bairro: VILA MENDONÇA **CEP:** 16.015-050
UF: SP **Município:** ARACATUBA
Telefone: (18)3636-3234 **Fax:** (18)3636-3203 **E-mail:** cep.foa@unesp.br

Considerações sobre os Termos de apresentação obrigatória:

Todos os termos foram apresentados de acordo com a resolução 466/12 do CNS.

Recomendações:

Não há.

Conclusões ou Pendências e Lista de Inadequações:

Relatório Final Aprovado.

Considerações Finais a critério do CEP:

Relatório Final Aprovado.

Este parecer foi elaborado baseado nos documentos abaixo relacionados:

Tipo Documento	Arquivo	Postagem	Autor	Situação
Envio de Relatório Final	Relatorio_final_anexos.pdf	10/08/2023 15:27:02	FERNANDA DE SOUZA E SILVA RAMOS	Postado
Envio de Relatório Final	Oficio_assinado.pdf	10/08/2023 15:27:27	FERNANDA DE SOUZA E SILVA RAMOS	Postado

Situação do Parecer:

Aprovado

Necessita Apreciação da CONEP:

Não

Documento assinado digitalmente
ANDRE PINHEIRO DE MAGALHAES BERTOZ
Data: 22/09/2025 17:43:09-0300
Verifique em https://validar.iti.gov.br

3

Assinado por:
André Pinheiro de Magalhães Bertoz
(Coordenador(a))

Endereço: JOSE BONIFACIO 1193
Bairro: VILA MENDONÇA **CEP:** 16.015-050
UF: SP **Município:** ARACATUBA
Telefone: (18)3636-3234 **Fax:** (18)3636-3203 **E-mail:** cep.foa@unesp.br
