# Supplementary material for: Longevity of different in-office treatments for dentin hypersensitivity: A 6-month randomized and parallel clinical trial
Source: PLoS One. 2026 Feb 17;21(2):e0342651. doi: 10.1371/journal.pone.0342651 (PMC12912554; doi:10.1371/journal.pone.0342651)
Supplement: S3 File — Original study protocol in Portuguese, submitted to the ethics committee. (PDF) [file pone.0342651.s003.pdf]

Efeito de diferentes tratamentos na hipersensibilidade dentinária: estudo clínico randomizado

Resumo

O objetivo deste estudo será investigar os efeitos de diferentes categorias de tratamentos para hipersensibilidade dentinária por meio de estudo clínico randomizado, utilizando os seguintes protocolos: verniz fluoretado (controle positivo, Duraphat –VF); solução com cerâmica bioativa cristalina (Biosilicato® - SS); sistema adesivo autocondicionante (Single Bond Universal – SB); verniz fotoativado bioativo (PRG - VB). Cento e noventa e dois dentes (48 por tratamento) com raiz exposta com hipersensibilidade dentinária (sem cavidade) serão tratados por meio de um estudo paralelo randomizado. O grau de sensibilidade dentinária será analisado por meio da escala visual analógica (VAS) e escala visual computadorizada (CoVAS), antes do tratamento (baseline) e 7, 15, 30 dias, 6 e 12 meses após o tratamento. Os resultados serão submetidos a testes estatísticos específicos. Espera-se que o presente estudo contribua para o conhecimento do mecanismo de ação de novos protocolos dessensibilizantes, bem como para a obtenção de um protocolo clínico inovador e eficaz para a hipersensibilidade dentinária.

Palavras chaves: Desgaste dos dentes; Dessensibilizantes dentinários; Erosão dentária; Estudo clínico

1. Introdução e justificativa

A hipersensibilidade dentinária (HD) é definida como uma dor aguda de curta duração causada por estímulos térmicos, táteis, químicos ou osmóticos.[1,2,3] Dentre os mecanismos que explicam a HD, a teoria hidrodinâmica pressupõe que os estímulos externos movimentam o fluído do interior dos túbulos dentinários e geram contração e distensão dos processos odontoblásticos, estimulando as fibras nervosas da interface dentina-polpa.[4] Essa condição está intimamente relacionada à exposição radicular, com presença ou não de lesões cervicais não cariosas (LCNC). Os tratamentos propostos incluem procedimento cirúrgico de cobertura radicular, realização de restaurações adesivas em casos de cavitação ou aplicação de produtos dessensibilizantes quando o procedimento cirúrgico ou a restauração não forem os tratamentos de eleição.[5] Os produtos dessensibilizantes agem por meio da obliteração dos túbulos dentinários ou redução da excitabilidade nervosa; entretanto, devido a etiologia multifatorial da HD, a eficácia é reduzida a longo prazo.[5,6] Atualmente, a literatura apresenta três categorias de produtos para o tratamento da HD: vernizes fluoretados, soluções experimentais de produtos bioativos e produtos com agentes fotopolimerizáveis.[5]

Os vernizes fluoretados caracterizam grande parte dos protocolos utilizados, uma vez que possuem a capacidade de reduzir ou bloquear a movimentação dos fluidos nos túbulos dentinários através da formação de precipitados de cálcio-fósforo, contudo, pode apresentar limitação quanto a seus efeitos a longo prazo, por essa razão, tem-se buscado outros produtos com a finalidade de prologar os efeitos dessensibilizantes. [7,8,9]

As soluções experimentais com objetivos bioativos, outra categoria testada para o tratamento da HD, incluem materiais que apresentam uma estrutura mais próxima da

porção mineral dos dentes.[10] Dentre eles há soluções com cerâmica bioativa cristalina, os biosilicatos, que foram propostos com objetivo de promover a remineralização de tecidos duros pela precipitação de fosfato de cálcio e formação de hidroxiapatita. [10,11,12] Tal propriedade atribui a esses materiais um potencial efeito obliterador nos túbulos dentinários, além de remineralização e impedimento de desmineralização em dentina.[11,12,13]

Considerando a categoria dos produtos com agentes fotopolimerizáveis, os sistemas adesivos universais garantem uma opção de tratamento por promoverem o selamento dos túbulos dentinários e formação de uma camada híbrida, neutralizando o mecanismo hidrodinâmico da hipersensibilidade.[17,18] Além desse material, recentemente foi lançado no mercado odontológico um produto que alia todas as composições supracitadas, sendo um verniz fotopolimerizável com partículas de vidro pré-reagido de superfície que permite a liberação de flúor. Esta tecnologia bioativa permite que as partículas de vidro multifuncionais aprisionadas na matriz poliácida, que liberem também outros íons como estrôncio, borato, alumínio, silicato e sódio. Sendo assim, ocorre a neutralização dos ácidos oriundos da alimentação e remineralização dos tecidos, aliando-se à obliteração por meio de monômeros fotopolimerizáveis.[19]

Marto et al. (2019)[20] por meio de uma metanálise que avaliou estudos clínicos randomizados com diversos tratamentos para a HD, concluíram que apenas os tratamentos em consultório são eficazes na redução imediata da HD, podendo manter sua eficácia ao longo do tempo; no entanto, mais protocolos de tratamento devem ser estudados, aumentando os tempos de análise.

Diante do exposto, torna-se oportuno a realização de um estudo que avalie novos protocolos para reduzir a HD utilizando metodologias por meio de estudo clínico randomizado.

2. Objetivos

O objetivo do estudo será avaliar a influência de diferentes protocolos para o tratamento da hipersensibilidade dentinária. As hipóteses nulas a serem testadas serão: a) não haverá diferença estatisticamente significante em relação à sensibilidade dentinária entre os protocolos dessensibilizantes em cada tempo de avaliação. b) não haverá diferença estatisticamente significante entre os tempos de avaliação quando um mesmo protocolo dessensibilizante for avaliado.

Este projeto de pesquisa será enviado ao comitê de ética local e será registrado em base de dados de estudo clínico (REBEC). O estudo apresenta-se descrito conforme a recomendação do Consort.[21]

3. Materiais e métodos

3.2.1 Delineamento Experimental

Este será um estudo clínico paralelo, prospectivo, randomizado. Somente os voluntários não saberão em qual grupo estarão incluídos (protocolo simples cego).

Inicialmente será planejado uma amostra de 192 dentes com dentina radicular exposta hipersensível, com 48 dentes por grupo. Os fatores de estudo serão: (1) tratamento dessensibilizante em 4 níveis (Tabela 1): verniz fluoretado (controle positivo,

Duraphat– VF); solução com cerâmica bioativa cristalina (Biosilicato® - SS); sistema adesivo autocondicionante (Single Bond Universal – SB); verniz fluoretado fotopolimerizável (VL); e (2) tempos de análise sendo inicial (previamente aos tratamentos, 7, 15, 30 dias, 6 e 12 meses após). As variáveis de respostas serão análise da sensibilidade por meio das escalas visual analógica (VAS) e visual computadorizada (CoVAS).

3.2.2 Cálculo da amostra e Seleção dos pacientes

O cálculo do tamanho da amostra foi realizado, baseado em artigo,[22] usando o software Sigma Plot 12.0, com esperada diferença entre as médias do nível de hipersensibilidade da dentina cervical medida pela escala visual analógica (VAS) de 0,210 após seis meses.[22] Os detalhes do teste foram: nível de significância (α) = 0.05; poder do teste (1-β) = 0,80; dropout (β)=0,2. O número da amostra final foi de 48 dentes por grupo. Serão selecionados pacientes na clínica de graduação da FOA-UNESP, entre 20-70 anos que possuam no mínimo uma exposição radicular totalizando 192 dentes com HD em exposições radiculares não cavitadas (sem necessidade de restauração), independentemente da localização na arcada dentária. Os dentes serão divididos em três grupos (n = 48 dentes) com nível de significância de 5%. [23,24] Os critérios de elegibilidade estão descritos na Tabela 2.

Tabela 1: Produtos a serem utilizados no estudo e sua composição segundo os fabricantes

Material	Fabricante	Composição*	Aplicação*
**Duraphat (VF)**	Colgate-Palmolive Company	NaF 5% (22.600 ppm), colofônia, álcool etílico, goma-laca, mástica, sacarina, aroma, cera branca de abelha.	Uma fina camada será aplicada com microbrush descartável sobre a superfície limpa e seca, permanecendo estável por 10 minutos.
**Biosilicato® (SS)**	Vitrovita, Instituto de Inovação em Vitrocerâmicos	Solução 1:10 de pó (1-10 µm) e água destilada Pó: P_2; O_5-Na_2; O-CaO-SiO_2	
**Single Bond Universal (SB)**	3M ESPE, Produtos Odontológicos	Álcool etílico; BISGMA; Sílica tratada com silano; HEMA; Copolímero de ácido acrílico e itacônico; UDMA; Água; Hexafluorofosfato de difeniliodônio	Uma fina camada será aplicada com microbrush descartável sobre a superfície limpa e seca. O produto será friccionado por 20 s previamente evaporação do solvente por 5 s. Em seguida, será fotoativado por 10 s.
**PRG – Barrier Coat (VB)**	Shofu INC.	S S-PRG (3,0 µm) à base de água destilada com fluoro-boro aluminossilicato; monômero de ácido metacrílico; monômero de ácido fosfônico; monômero de ácido metacrilico Bis-MPEPP; monômero de ácido carboxílico; TEGDMA; iniciador de reação e outros.	Será adicionado uma gota do ativador no casulo da base. A mistura será aplicada, com pincel próprio, em uma fina camada sobre a superfície permanecendo por 3 s previamente a fotoativação por 10 s.

Abreviaturas: TMP (trimetafosfato de sódio nanoparticulado) TEGDMA (dimetilacrilato de etileno glicol); BISGMA (Dimetacrilato de éter diglicidílico do bisfenol A); HEMA (Metacrilato de 2-hidroxietilo); UDMA (1,3 dimetacrilato de glicerol; Dimetacrilato de diuretan; Bis-MPEPP (bisphenol A polyethoxy methacrylate)

*De acordo com o fabricante§ de Melo Alencar, et. al. (2019)[7] § Fujimoto, et. al. (2010)[25]

Tabela 2: Critérios de inclusão e exclusão de pacientes

**Critérios de Inclusão**	**Critérios de Exclusão**
- Idade entre 20 e 70 anos, independente do sexo<br>*- Boa saúde, sem história de alergias a produtos odontológicos*<br>*- Presença de no mínimo 1 exposição radicular não cavitada ou com início de cavitação (até 1mm de profundidade, sem necessidade de restauração), que apresentem sensibilidade* de pelo menos grau 3 (VAS) *ao jato de ar em uma distância de 10cm*<br>*- Ausência de doença periodontal ativa*	*- Gestantes, lactantes ou fumantes*<br>*- Presença de lesões de cárie ativas*<br>*- Uso de agentes dessensibilizantes nos últimos 6 meses*<br>*- Doença periodontal ativa e sem tratamento*<br>*-Uso de aparelho ortodôntico ou prótese parcial removível com grampo no dente a ser avaliado*<br>*- Hábitos parafuncionais e traumas oclusais*<br>*- Uso de analgésico/anti-inflamatório*

De acordo com as recomendações do Conselho Federal de Odontologia para atendimento durante a pandemia de COVID-19, o protocolo estabelecido será: todos os pacientes serão marcados em horários espaçados de forma a não gerar aglomerações; no ambiente de espera será disponibilizado solução de álcool 70% para higienização das mãos; não será permitido a permanência no local sem uso de máscaras; o ambiente clinico será higienizado a partir da região menos infectada para a mais infectada (alça do refletor, cadeira, mocho, superfície do carrinho auxiliar e equipo, respectivamente) entre cada atendimento; serão colocadas barreiras mecânicas de PVC nas regiões de contato manual do profissional (botões de acionamento mecânico, alças de refletores, encostos e braços da cadeira, encosto do mocho, canetas de alta e baixa rotação, corpo da seringa tríplice e pontas de sugadores); todos os instrumentais serão desinfectados com detergente enzimático e esterilizados.[26,27] Além disso, os pesquisadores usarão jaleco impermeável e descartável, gorro descartável, óculos de proteção, máscara cirúrgica (em procedimentos sem a geração de aerossóis), máscara PPF ou N95 (em procedimentos com a geração de aerossóis), protetor facial e luvas, os quais serão trocados a cada atendimento.[26,27]

Quanto a sensibilidade ao jato de ar nas exposições radiculares, será realizado isolamento relativo com roletes de algodão e os dentes adjacentes será isolado com fita isolante (Isotape, TDV Dental, Pomerode, Santa Catarina, Brasil).[18] O estímulo será por jato de ar, sendo a seringa tríplice posicionada a 1cm de distância da região cervical e terá duração máxima de 2 s. Imediatamente após, o paciente indicará o nível de sensibilidade pela escala VAS que consistirá em uma linha horizontal de 10 cm, a dor será identificada pelo paciente de 1 a 10 pontos, onde 0 significa "sem dor", 1-3 "dor leve", 4-6 "dor moderada" e 7-10 "dor severa". O paciente deverá marcar uma linha vertical cruzando a linha horizontal da escala a intensidade da HD sentida. Após esta marcação, a distância em *mm* das extremidades será medida com régua milimetrada.[35,38]

Serão selecionados os dentes com pelo menos grau 3 de sensibilidade pela escala VAS.

Após selecionados, cada paciente receberá um Termo de Consentimento Livre e Esclarecido com detalhamento do objetivo e dos procedimentos a serem realizados, que deverá ser assinado em duas vias uma vez que concorde em participar do estudo.

A escala CoVAS será então utilizada aplicando-se um constante estímulo, por meio de jato de ar, a uma distância de 10 mm, na face vestibular dos elementos dentários por 15 s. Durante este período, o paciente registrará a intensidade do desconforto em uma escala de 0 a 100, utilizando um potenciômetro controlado manualmente.[29]

Os dados das análises de sensibilidade VAS e CoVAS serão registrados (tempo inicial). Todos receberão escova dental macia e dentifrício sem efeito dessensibilizante para uso durante o tempo do experimento.

3.2.3 Avaliações prévias

Serão analisadas as condições do meio bucal do paciente, através do Índice de dentes permanentes cariados, perdidos e obturados (CPOD), placa visível (IPV) e sangramento gengival (ISG). Em seguida, com auxílio de uma sonda milimetrada, serão realizadas as mensurações de altura da exposição, considerando a distância entre a extremidade mais apical da junção cemento-esmalte e o ponto mais alto da gengiva marginal livre. Neste primeiro momento, os valores obtidos não serão classificados, somente após completar a amostra serão criados escores de acordo com as medidas obtidas.[30]

A altura da exposição radicular e o escore de sensibilidade pela escala VAS serão variáveis estratificadas no processo de randomização que será realizada como segue. O dente será considerado a unidade amostral. Os dentes elegíveis serão registrados em planilha Excel de acordo com a altura da exposição radicular e o escore médio da sensibilidade. Para haver uma distribuição homogênea desses dois fatores nos grupos de estudo, os dentes serão ordenados pelo escore de sensibilidade e divididos em dois conglomerados; um contendo as pontuações de sensibilidade mais baixas e o outro as pontuações mais altas. Esses dois conglomerados serão subdivididos de acordo com o tamanho da exposição radicular menor/maior, totalizando quatro conglomerados: (a) pontuações de sensibilidade mais baixas e tamanhos de exposições menores, (b) pontuações sensibilidades mais baixas e tamanhos de exposições maiores, (c) pontuações sensibilidade mais altas e exposições menores e (d) maiores escores de sensibilidade e exposições maiores. Este método estratificado de randomização foi baseado em outro estudo clínico paralelo.[31] Para uma maior facilidade metodológica esta estratificação será realizada a cada aproximadamente 48 dentes selecionados.

Nos casos em que os tratamentos experimentais não surtirem efeito sobre a sensibilidade dentinária, os pacientes receberam a aplicação do produto que obtiver o melhor resultado, de modo que danos maiores sejam evitados.

3.2.4 Procedimentos clínicos

Feito as avaliações prévias, um afastador bucal será posicionado, de modo a afastar lábios e bochecha. O dente que receberá o agente dessensibilizante será isolado com roletes de algodão e secos com jato de ar, com a umidade sendo controlada com o

auxílio de um sugador. O respectivo produto será aplicado em duas camadas com o auxílio de um micro aplicador, permanecendo por 10 minutos na superfície. Os produtos fotoativados serão ativados por 10 s (LED Radii-cal, SDI Brasil Indústria e Comércio LTDA, São Paulo, Brasil). Após isso, os roletes de algodão, bem como o afastador bucal serão removidos.

No final do estudo, nos casos em que os tratamentos experimentais não surtirem efeito sobre a sensibilidade dentinária, os pacientes receberão a aplicação do produto que obtiver o melhor resultado, de modo que danos maiores sejam evitados.

3.2.5 Análises

A intensidade da HD será novamente mensurada pelas escalas supramencionadas nos tempos de 7, 15, 30 dias e 6 e 12 meses.[22,32]

3.2.6 Análises estatísticas

Os resultados dos índices CPOD, IPV, ISG e altura das exposições radiculares serão expostos de forma descritiva. Para correlação entre os métodos para avaliação de sensibilidade utilizados no estudo (CoVAS e VAS), será realizado coeficiente de correlação de Spearman. As comparações entre os tratamentos no mesmo tempo e dentre os tempos em um mesmo tratamento serão analisadas por testes de igualdade de duas proporções com nível de significância a 5%.

4. Cronograma de execução

ETAPAS / *MESES*	A	B	C	D	E	F
1	X	X				
2	X	X				
3	X	X				
4	X	X				
5	X	X	X			
6	X		X	X		
7	X			X		
8	X			X		
9	X			X		
10	X			X		
11	X			X		
12	X			X	X	X
13	X				X	X
14	X					X
15	X					X
16	X					X

Etapa A – Levantamento bibliográfico em todos os estudos para busca na literatura sobre exposições radiculares, hipersensibilidade dentinária e tratamentos dessensibilizantes utilizados em estudos prospectivos.
Etapa B – Estudo *in vivo:* Seleção dos pacientes; avaliação inicial; aplicação dos produtos; avaliação 7, 15, 30 dias.
Etapa C – Estatística e análise dos dados até 30 dias
Etapa D – Estudo *in vivo*: avaliação de 6 e 12 meses; tabulação dos dados finais; análises estatísticas.
Etapa E - Redação do relatório científico final
Etapa F – Redação dos artigos científicos.

5-Referências

1- Que, K., Guo, B., Jia, Z., Chen, Z., et al. A cross-sectional study: non-carious cervical lesions, cervical dentine hypersensitivity and related risk factors. J Oral Rehabil, 40:24-32, 2013.

2- Zeola, F.L., Soares, P.V., Cunha-Cruz, J. Prevalence of dentin hypersensitivity: systematic review and meta-analysis. J Dent, 81:1-6, 2019.

3- Felix, J., Ouanounou, A. Dentin hypersensitivity: Etiology, diagnosis, and management. Compend Contin Educ Dent, 40(10):653-657, 2019.

4- Chung, G., Jung, S.J., Oh, S.B. Cellular and molecular mechanisms of dental nociception. J Dent Res, 92:948-55, 2013.

5- Peumans, M., Politano, G., Van Meerbeek, B. Treatment of noncarious cervical lesions: When, why and how. Int J Esthet Dent, 15:16-42, 2020.

6- Yilmaz, H.G., Kurtulmus-Yilmaz, S., Cengiz, E. Long-term effect of diode laser irradiation compared to sodium fluoride varnish in the treatment of dentine hypersensitivity in periodontal maintenance patients: A randomized controlled clinical study. Photomed Laser Surg, 29: 721-5, 2011.

7- de Melo Alencar, C., de Franca Leite, K.L., Ortiz, M.I.G., Magno, M.B., et al. Morphological and chemical effects of in-office and at-home desensitising agents containing sodium fluoride on eroded root dentin. Arch Oral Biol, 110: 104619, 2019.

8- Kim, H.N., Kim, J.B., Jeong, S.H. Remineralization effects when using different methods to apply fluoride varnish in vitro. J Dent Sci, 13:360-366, 2018.

9- Pichaiaukrit, W., Thamronganankul, N., Siralertmukul, K., Swasdison S. Fluoride varnish containing chitosan demonstrated sustained fluoride release. Dent Mater J, 38:1036-1042, 2019

10- Renno, A.C.M, Bossini, P.S.B, Crovace, M.C., Rodrigues, A.C.M, Zanotto, E.D., Parizotto, N.A. Characterization and in vivo biological performance of biosilicate. Biomed Res Int, 2013:141427, 2013.

11- Pintado-Palomino, K., Tirapelli, C. The effect of home-use and in-office bleaching treatments combined with experimental desensitizing agents on enamel and dentin. Eur J Dent, 9:66-73, 2015.

12- Tirapelli, C., Panzeri, H., Lara, E.H.G., Soares, R.G., Peitl, O., Zanotto, E.D. The effect of a novel crystallized bioactive glass-ceramic powder on dentine hypersensitivity: a long-term clinical study. J Oral Rehabil, 38(4):253-262, 2011

13- Tirapelli, C., Panzeri, H., Soares, G.R., Peitl, O., Zanotto, D.E. A novel bioactive glass-ceramic for treating dentin hypersensitivity. Braz Oral Res, 24(4):381-7, 2010.

14- Abbarin, N., Miguel, S.S., Holcroft, J., Iwasaki, K., Ganss, B. The enamel protein amelotin is a promoter of hydroxyapatite mineralization. J Bone Miner Res, 30(5):775–785, 2015.

15- Ikeda, Y., Neshatian, M., Holcroft, J., Ganss, B. The enamel protein ODAM promotes mineralization in a collagen matrix. Connect Tissue Res, 59(1):62-66, 2018.

16- Fouillen, A., Neves, J.S., Mary, C., Castonguay, J.D., Moffatt, P., Baron, C., Nanci, A. Interactions of AMTN, ODAM and SCPPPQ1 proteins of a specialized basal lamina that attaches epithelial cells to tooth mineral Sci Rep, 24(7):46683, 2017.

17- Patil, S.A., Naik, B.D., Suma, R. Evaluation of three different agents for in-office treatment of dentinal hypersensitivity: A controlled clinical study. Indian J Dent Res, 26(1):38-42, 2015.
18- Askari, M., Yazdani, R. Comparison of two desensitizing agents for decreasing dentin hypersensitivity following periodontal surgeries: A randomized clinical trial. Quintessence Int, 50(4):320-329, 2019.
19- Ravishankar, P., Viswanath, V., Archana, D., Keerthi, V., et al. The effect of three desensitizing agents on dentin hypersensitivity: A randomized, split-mouth clinical trial. Indian J Dent Res, 29:51-55, 2018.
20- Marto, C.M., Paula, A.B., Nunes, T., Pimenta, M., Abrantes, A.M., Pires, A.S., Laranjo, M., Coelho, A., Donato, H., Botelho, M.F., Ferreira, M.M., Carrilho, E. Evaluation of the efficacy of dentin hypersensitivity treatments-A systematic review and follow-up analysis. J Oral Rehabil, 46(10):952-990, 2019
21- Schulz, K.F., Altman, D.G., Moher, D. CONSORT 2010 statement: updated guidelines for reporting parallel group randomized trials. PLoS Med 7, e1000251, 2010.
22- Moura, G.F., Zeola, L.F., Silva, M.B., Sousa, S.C., Guedes, F.R., Soares, P.V. Four-session protocol effectiveness in reducing cervical dentin hypersensitivity: a 24-week randomized clinical trial. Photobiomodul Photomed Laser Surg, 37(2):177-123, 2019.
23- Ritter, A.V., Dias, W.L., Miguez, P., Caplan, D.J., Swift, E.J. Jr. Treating cervical dentin hypersensitivity with fluoride varnish: a randomized clinical study. J Am Dent Assoc, 137:1013-20, 2006.
24- Sgreccia P.C., Barbosa R.E.S., Damé-Teixeira N., Garcia F.C.P. Low-power laser and potassium oxalate gel in the treatment of cervical dentin hypersensitivity – a randomized clinical trial. Clin Oral Investig. https://10.1007/s00784-020-03311- 7 2020.
25- Fujimoto Y, Iwasa M, Murayama R, Miyazaki M, Nagafuji A, Nakatsuka T. Detection of ions released from S-PRG fillers and their modulation effect. Dent Mater J, 2010;29(4):392-397.
26- Peng, X., Xu, X., Li, Y., Cheng, L., Zhou, X., Ren, B. Transmission routes of 2019-nCoV and controls in dental practice. Int J Oral Sci, 12: https://10.1038/s41368-020-0075-9, 2020.
27- Kampf, G., Todt, D., Pfaender, S., Steinmann, E. Persistence of coronaviruses on inanimate surfaces and their inactivation with biocidal agents. J Hosp Infect, 104:246-251, 2020.
28- Rahal, V., Gallinari, M.O., Barbosa, J.S., Martins-Junior, R.L., dos Santos, P.H., Cintra, L.T.A., Briso, A.L.F. Influence of skin cold sensation threshold in the occurrence of dental sensitivity during dental bleaching: a placebo controlled clinical trial. J Appl Oral Sci, 26:e20170043, 2018.
29- Briso, A.L.F., Rahal, V., Azevedo, F.A., Gallinari, M.O., Gonçalves, R.S., Frascino, S.M.B, Santos, P.H.D., Cintra, L.T.A. Neurosensory analysis of tooth sensitivity during at-home dental bleaching: a randomized clinical trial. J Appl Oral Sci, 26:e20170284, 2018.
30- Shinohara, M.S., Carvalho, P.R.M., Neves Marcon, L., Gonçalves, D.F.M., Ramos, F.S.S., Fagundes, T.C. Randomized clinical trial of different adhesion strategies in noncarious cervical lesion restorations: 1-year follow-up. Quintessence Int, 51(5):352-

363, 2020.
31- Menezes-Silva, R., Velasco, S.R.M, Bastos,R.S, Molina, G., Honório, H.M., Frencken, J.E., Navarro, M.F.L. Randomized clinical trial of class II restoration in permanent teeth comparing ART with composite resin after 12 months. Clin Oral Investig, 23(9):3623-3635, 2019.
32- Sivaramakrishnan, G., Sridharan, K. Fluoride varnish versus glutaraldehyde for hypersensitive teeth: a randomized controlled trial, meta-analysis and trial sequential analysis. Clin Oral Investig, 23(1):209-220, 2019.
