# Supplementary material for: Longevity of different in-office treatments for dentin hypersensitivity: A 6-month randomized and parallel clinical trial
Source: PLoS One. 2026 Feb 17;21(2):e0342651. doi: 10.1371/journal.pone.0342651 (PMC12912554; doi:10.1371/journal.pone.0342651)
Supplement: S4 File — Translated ethics committee approval document in Portuguese. (PDF) [file pone.0342651.s004.pdf]

UNESP - SCHOOL OF DENTISTRY - ARAÇATUBA CAMPUS / SÃO PAULO STATE UNIVERSITY "JÚLIO DE MESQUITA FILHO"

CONSOLIDATED OPINION OF THE RESEARCH ETHICS COMMITTEE (CEP)

Researcher: FERNANDA DE SOUZA E SILVA RAMOS

Research Title: Effect of different protocols in the treatment of dentin hypersensitivity: in situ and in vivo evaluation

Proposing Institution: School of Dentistry of Araçatuba Campus - UNESP

Version: 1

CAAE: 30122220.1.0000.5420

Opinion Number: 3.988.387

Objective: To investigate the effects of applying different desensitizing agents on hypersensitive root exposures. Methods: For the in situ study, 72 bovine dentin specimens with open dentinal tubules will be subdivided into the following groups: fluoride varnish (Duraphat, Colgate, Colgate-Palmolive Company); 5% NaF varnish with 5% sodium trimetaphosphate nanoparticles (TMP); and a light-cured varnish (PRG Barrier Coat, Shofu INC.). After initial microhardness analysis for block selection, the specimens will receive an acid-resistant varnish layer and will be subjected to erosive/abrasive cycles. At the end of the cycles, specimens will be analyzed by profilometry and internal hardness. Volunteer cooperation will be assessed through the TheraMon® microsensor. For the in vivo study, 45 patients with at least two teeth with exposed root dentin, non-cavitated and with dentin hypersensitivity, will be selected, totaling 90 teeth, which will be divided according to the in situ groups. Applications will be performed and evaluated at the following times: baseline, after 7, 15, 30 days and 6 months. Qualitative analyses will be carried out by visual and computerized analog scale (VAS and CoVAS). Quantitative analysis will be performed using a neurosensory analysis device. After data collection, results will be subjected to appropriate statistical tests.

Funding: Self-funded

Contact: JOSE BONIFACIO 1193 - VILA MENDONÇA
ZIP code: 16.015-050
Phone: (18) 3636-3200 E-mail: andrebertoz@foa.unesp.br
State: SP Municipality: ARAÇATUBA Fax: (18) 3636-3332

Clinical relevance: The present study has the potential to obtain an innovative and effective clinical protocol for the treatment of dentin hypersensitivity.

Primary Objective:

The objective of the in situ study will be to evaluate profilometry and internal hardness of dentin after applying three desensitizing products (Duraphat Fluoride Varnish, 5% NaF Varnish with 5% TMP nanoparticles, PRG Barrier Coat) subjected to erosive/abrasive challenge. The objective of the in vivo study will be to quantify dentin sensitivity after applying the same three products using the visual and computerized analog scale (VAS and CoVAS), and a neurosensory analysis device.

Study Objective:

Risks: Minimal risks, inherent to any routine dental treatment.

Benefits: Patients will have their teeth treated for sensitivity. They will also be referred to other disciplines if another type of dental treatment is needed.

Risk and Benefit Assessment:

The research is suitable for implementation.

Comments and Considerations on the Research:

All terms were added in accordance with CNS Resolution 466/12.

Considerations on mandatory submission terms: None.

Recommendations: The research is suitable for implementation.

Conclusions or Pending Issues and List of Inadequacies:

We emphasize that, in accordance with CNS Resolution 466, of 12/12/2012 (title X, section X.1., art. 3, item b, e, title XI, section XI.2., item d), there is a need to present semiannual reports, with the first report due by 10/01/2020.

ARACATUBA, April 24, 2020

Aldiéris Alves Pesqueira

(Coordinator)

This opinion was prepared based on the following documents:

Basic Project Information - PB_INFORMAÇÕES_BÁSICAS_DO_PROJETO_1529447.pdf - Posted: 03/20/2020 11:07:21 - Status: Accepted

ICF / Assent Terms / Justification of Absence - TCLE.pdf - Posted: 03/20/2020 10:48:42 - Author: FERNANDA DE SOUZA E SILVA RAMOS - Status: Accepted

Detailed Project / Investigator's Brochure - ProjetodePesquisa.pdf - Posted: 03/20/2020 10:48:24 - Author: FERNANDA DE SOUZA E SILVA RAMOS - Status: Accepted

Cover Sheet - FolhaRosto.pdf - Posted: 03/20/2020 10:46:30 - Author: FERNANDA DE SOUZA E SILVA RAMOS - Status: Accepted

Opinion Status: Approved

Requires CONEP Review: No
