# Supplementary material for: Longevity of different in-office treatments for dentin hypersensitivity: A 6-month randomized and parallel clinical trial
Source: PLoS One. 2026 Feb 17;21(2):e0342651. doi: 10.1371/journal.pone.0342651 (PMC12912554; doi:10.1371/journal.pone.0342651)
Supplement: S5 File — Translated final report submitted to the ethics committee in Portuguese. (PDF) [file pone.0342651.s005.pdf]

UNESP - SCHOOL OF DENTISTRY - ARAÇATUBA CAMPUS / SÃO PAULO STATE UNIVERSITY "JÚLIO DE MESQUITA FILHO"

CONSOLIDATED OPINION OF THE RESEARCH ETHICS COMMITTEE (CEP)

Researcher: FERNANDA DE SOUZA E SILVA RAMOS

Research Title: Effect of different treatments on dentin hypersensitivity: cytotoxicity evaluation and randomized clinical trial

Proposing Institution:

Version: 4

CAAE: 30122220.1.0000.5420

Opinion Number: 6.267.262

Submission of the final report of the research activities: Effect of different treatments on dentin hypersensitivity: cytotoxicity evaluation and randomized clinical trial.

Type of Notification Submitted: Final Report

Objective of the Notification: Minimal risk.

Risk-Benefit Assessment: Final report approved.

Comments and Considerations regarding the Notification: Submission of Final Report

Attached I forward the final report of the project entitled "Effect of different treatments on dentin hypersensitivity: cytotoxicity evaluation and randomized clinical trial."

Consolidated Opinion Issued: 10/08/2023

Funding: Self-funded

Contact: JOSE BONIFACIO 1193 - VILA MENDONÇA

Postal code: 16.015-050

Phone: (18) 3636-3234   E-mail: cep.foa@unesp.br

State: SP   Municipality: ARAÇATUBA   Fax: (18) 3636-3203

All terms were presented in accordance with National Health Council (CNS) Resolution 466/12.

Considerations regarding the mandatory presentation terms: None.

Recommendations: Final Report Approved.

Conclusions or Pending Issues and List of Deficiencies: Final Report Approved.

Final considerations at the CEP's discretion:

ARACATUBA, August 29, 2023

gov.br Documento assinado digitalmente
ANDRE PINHEIRO DE MAGALHAES BERTOZ
Data: 22/09/2025 17:39:29-0300
Verifique em https://validar.iti.gov.br

André Pinheiro de Magalhães Bertoz

(Coordinator)

This opinion was prepared based on the documents listed below:

Document Type: Submission of Final Report - Relatorio_final_anexos.pdf - Posted: 10/08/2023 15:27:02 - Author: FERNANDA DE SOUZA E SILVA RAMOS

Document Type: Submission of Final Report - Oficio_assinado.pdf - Posted: 10/08/2023 15:27:27 - Author: FERNANDA DE SOUZA E SILVA RAMOS

Opinion Status: Approved

Requires CONEP Review: No

Contact: JOSE BONIFACIO 1193 - VILA MENDONÇA

Postal code: 16.015-050

Phone: (18) 3636-3234   E-mail: cep.foa@unesp.br

State: SP   Municipality: ARAÇATUBA   Fax: (18) 3636-3203
