# Supplementary material for: Longevity of different in-office treatments for dentin hypersensitivity: A 6-month randomized and parallel clinical trial
Source: PLoS One. 2026 Feb 17;21(2):e0342651. doi: 10.1371/journal.pone.0342651 (PMC12912554; doi:10.1371/journal.pone.0342651)
Supplement: S6 File — Translated study protocol in Portuguese, submitted to the ethics committee. (PDF) [file pone.0342651.s006.pdf]

Effect of Different Treatments on Dentin Hypersensitivity: A Randomized Clinical Trial

Abstract
The objective of this study is to investigate the effects of different categories of treatments for dentin hypersensitivity through a randomized clinical trial, using the following protocols: fluoride varnish (positive control, Duraphat – VF); solution containing crystalline bioactive ceramic (Biosilicate® – SS); self-etch adhesive system (Single Bond Universal – SB); and bioactive light-cured varnish (PRG – VB). A total of 192 teeth (48 per treatment) with exposed root surfaces presenting dentin hypersensitivity (without cavitation) will be treated in a randomized parallel study design. The degree of dentin hypersensitivity will be evaluated using the Visual Analog Scale (VAS) and the Computerized Visual Analog Scale (CoVAS), at baseline and after 7, 15, and 30 days, as well as 6 and 12 months post-treatment. Data will be subjected to appropriate statistical analyses. This study is expected to contribute to the understanding of the mechanisms of action of new desensitizing protocols and to provide an innovative and effective clinical approach for the management of dentin hypersensitivity.

Keywords: Tooth Wear; Dentin Desensitizing Agents; Dental Erosion; Clinical Trial

1. Introduction and Justification

Dentin hypersensitivity (DH) is defined as a short, sharp pain arising from thermal, tactile, chemical, or osmotic stimuli.[1,2,3] Among the mechanisms proposed to explain DH, the hydrodynamic theory assumes that external stimuli promote fluid movement within the dentinal tubules, resulting in contraction and expansion of odontoblastic processes, thereby stimulating nerve fibers at the dentin–pulp interface.[4] This condition is closely associated with root exposure, with or without the presence of non-carious cervical lesions (NCCLs). Treatment options include surgical root coverage procedures, adhesive restorations in cases of cavitation, or the application of desensitizing agents when surgery or restorative procedures are not the treatments of choice.[5] Desensitizing agents act either by obliterating dentinal tubules or by reducing nerve excitability; however, due to the multifactorial etiology of DH, their long-term effectiveness is limited.[5,6] Currently, the literature identifies three main categories of products for the treatment of DH: fluoride varnishes, experimental bioactive solutions, and light-cured agents.[5]

Fluoride varnishes account for a large portion of the protocols used, as they are capable of reducing or blocking fluid movement within dentinal tubules through the formation of calcium-phosphate precipitates. However, their long-term effectiveness may be limited, which has encouraged the development of alternative products designed to prolong the desensitizing effect.[7,8,9]

Experimental solutions with bioactive potential, another category tested for DH management, include materials with a structure more closely resembling the mineral portion of teeth.[10] Among these are crystalline bioactive ceramic solutions, such as biosilicates, which were proposed to promote hard tissue remineralization by calcium phosphate precipitation and hydroxyapatite formation.[10,11,12] This property provides these materials with potential for tubule occlusion, dentin remineralization, and prevention of demineralization.[11,12,13]

Considering the category of light-cured agents, universal adhesive systems provide a treatment option by sealing dentinal tubules and forming a hybrid layer, thereby neutralizing the hydrodynamic mechanism of hypersensitivity.[17,18] In addition, a more recent product has been introduced to the dental market: a light-cured varnish containing surface pre-reacted glass particles capable of releasing fluoride. This bioactive technology allows multifunctional glass particles trapped within the polyacid matrix to release other ions, including strontium, borate, aluminum, silicate, and sodium. As a result, the product contributes to neutralizing dietary acids and remineralizing tissues, while also promoting tubule occlusion through polymerizable monomers.[19]

Marto et al. (2019)[20], in a meta-analysis evaluating randomized clinical trials with different DH treatments, concluded that only in-office treatments are effective in producing immediate reduction of hypersensitivity and may maintain their effect over time. However, further treatment protocols should be studied, with extended follow-up periods.

Therefore, conducting a study to evaluate new protocols for the reduction of DH through randomized clinical trial methodologies is warranted

.

2. Objectives

The objective of this study is to evaluate the influence of different protocols for the treatment of dentin hypersensitivity. The null hypotheses to be tested are as follows: a) there will be no statistically significant difference in dentin hypersensitivity among the desensitizing protocols at each evaluation time point; b) there will be no statistically significant difference across evaluation time points when the same desensitizing protocol is assessed.

This research project will be submitted to the local ethics committee and registered in a clinical trial database (REBEC). The study is described in accordance with the CONSORT recommendations.[21]

3. Materials and Methods

3.1 Experimental Design

This will be a parallel, prospective, randomized clinical trial. Only the volunteers will remain blinded to the allocation group (single-blind design).

A sample of 192 teeth with exposed hypersensitive root dentin will be planned, with 48 teeth per group. The study factors will be: (1) desensitizing treatment at four levels

(Table 1): fluoride varnish (positive control, Duraphat – VF); solution containing crystalline bioactive ceramic (Biosilicate® – SS); self-etch adhesive system (Single Bond Universal – SB); and light-cured fluoride varnish (VL); and (2) evaluation times: baseline (prior to treatments), and 7, 15, and 30 days, as well as 6 and 12 months post-treatment. The response variables will be hypersensitivity assessment through the Visual Analog Scale (VAS) and the Computerized Visual Analog Scale (CoVAS).

3.2 Sample Size Calculation and Patient Selection

Sample size was calculated based on a published study[33] using Sigma Plot 12.0 software, with an expected mean difference in cervical dentin hypersensitivity levels (measured by VAS) of 0.210 after six months.[22] Test details: significance level (α) = 0.05; test power ($1-\beta$) = 0.80; dropout (β) = 0.2. The final sample size was determined as 48 teeth per group.

Patients will be recruited from the undergraduate clinic of FOA-UNESP, aged between 20 and 70 years, with at least one root exposure, totaling 192 teeth with DH in non-cavitated root exposures (not requiring restoration), regardless of location in the dental arch. Teeth will be divided into three groups (n = 48 teeth each) with a 5% significance level.[23,24] Eligibility criteria are described in Table 2.

Table 1. Products to be used in the study and their composition according to the manufacturers

Material	Manufacturer	Composition*	Application*
**Duraphat (VF)**	Colgate-Palmolive Company®	NaF 5% (22,600 ppm); colophony; ethanol; shellac; mastic; saccharin; flavoring; white wax; beeswax	A thin layer will be applied with a microbrush on the clean and dry surface; stable for 10 minutes.
**Biosilicate® (SS)**	Vitrovita, Institute of Innovation in Vitroceramics®	Solution 1:10 of P (1–10 μm) and distilled water. Elements: P_2O_5; Na_2O; CaO; SiO_2	A thin layer will be applied with a microbrush on the clean and dry surface. The solvent will be evaporated for 5 s and then rubbed for 20 s. Finally, light curing will be performed for 10 s.
**Single Bond Universal (SB)**	3M ESPE, Dental Products®	Ethanol; BISGMA; Silane treated with methacrylate; HEMA; Copolymer of acrylic and itaconic acid; UDMA; Water; Dipentaerythritol pentaacrylate phosphate; Initiator	A thin layer will be applied with a microbrush on the clean and dry surface. The solvent will be evaporated for 5 s and then rubbed for 20 s. Finally, light curing will be performed for 10 s.
**PRG Barrier Coat (VB)**	Shofu INC.®	S-PRG (3.0 μm) based on distilled water; fluoroboroaluminosilicate glass; methacrylic acid monomer; phosphonic acid monomer; Bis-MPEPP; carboxylic acid monomer; TEGDMA; initiator and others	A drop of activator will be mixed in the capsule. The mixture will be applied with a disposable brush in a thin layer on the surface, dried for 3 s, and then light cured.

Abbreviations: TMP (sodium trimetaphosphate nanoparticle); TEGDMA (triethylene glycol dimethacrylate); BISGMA (bisphenol A diglycidyl ether dimethacrylate); HEMA (2-hydroxyethyl methacrylate); UDMA (urethane dimethacrylate); Bis-MPEPP (bisphenol A polyethoxy methacrylate).

*According to manufacturers: de Melo Alencar et al. (2019)[7]; Fujimoto et al. (2010)[25].

Table 2. Inclusion and exclusion criteria for patients

Inclusion Criteria	Exclusion Criteria
-Age between 20–70 years, regardless of sex<br>*-Good general health; no history of allergies to dental products*<br>*-Presence of at least one non-cavitated root exposure (≥1 mm depth, not requiring restoration) presenting dentin hypersensitivity of at least grade 3 (VAS) to air-jet stimulation at 10 cm distance*<br>*-Absence of active periodontal disease*	*-Pregnant, lactating, or smoking patients*<br>*-Presence of active caries lesions*<br>*-Use of desensitizing agents in the last 6 months*<br>*-Active periodontal disease without treatment*<br>*-Use of orthodontic appliances or removable prostheses with clasps on the evaluated tooth*<br>*-Parafunctional habits and occlusal trauma*<br>*-Use of analgesics or anti-inflammatory drugs*

Following the guidelines of the Federal Council of Dentistry for clinical care during the COVID-19 pandemic, the following protocol will be adopted: patients will be scheduled at staggered times to avoid crowding; 70% alcohol solution will be available in waiting areas for hand hygiene; face masks will be mandatory; the clinical environment will be disinfected between appointments from the least contaminated to the most contaminated areas (reflector handle, chair, stool, assistant cart surface, and dental unit, respectively); PVC barriers will be applied to contact surfaces (mechanical buttons, reflector handles, chair backrests and armrests, stool backrest, handpieces, three-way syringe body, and suction tips); all instruments will be disinfected with enzymatic detergent and sterilized.[26,27]

Researchers will wear impermeable disposable gowns, disposable caps, protective goggles, surgical masks (for non-aerosol procedures), PPF or N95 masks (for aerosol-generating procedures), face shields, and gloves, all changed after each appointment.[26,27] Regarding air-jet sensitivity testing in root exposures, relative isolation with cotton rolls will be used, and adjacent teeth will be covered with insulating tape (Isotape, TDV Dental, Pomerode, Santa Catarina, Brazil).[18] The stimulus will consist of an air jet delivered by a three-way syringe positioned 1 cm from the cervical region for a maximum of 2 s. Immediately afterward, patients will indicate sensitivity level on the VAS, which consists of a 10 cm horizontal line where pain is scored from 0 to 10 points: 0 = no pain, 1–3 = mild pain, 4–6 = moderate pain, and 7–10 = severe pain. Patients will mark a vertical line across the horizontal scale to indicate DH intensity. The distance in mm from the zero endpoint will be measured with a millimeter ruler.[24,28] Teeth with at least grade 3 sensitivity on the VAS will be included.

After selection, each patient will receive and sign an informed consent form (in duplicate), detailing study objectives and procedures.

The CoVAS will then be used by applying a constant air-jet stimulus, 10 mm from the vestibular surface of the teeth, for 15 s. During this period, the patient will record discomfort intensity on a 0–100 scale using a manually controlled potentiometer.[29] Sensitivity analyses (VAS and CoVAS) will be recorded at baseline. All participants will receive a soft-bristle toothbrush and a non-desensitizing toothpaste for use throughout the study.

3.3 Baseline Assessments

Patients' oral conditions will be assessed through the Decayed, Missing, and Filled Teeth Index (DMFT), Visible Plaque Index (VPI), and Gingival Bleeding Index (GBI). Next, with a millimeter probe, root exposure height will be measured as the distance between the apical limit of the cemento-enamel junction and the highest point of the free gingival margin. At this stage, the values will not be classified; scores will be established only after the complete sample has been obtained.[30]

Root exposure height and sensitivity scores by VAS will be stratification variables in the randomization process. The tooth will be considered the sampling unit. Eligible teeth will be entered into an Excel spreadsheet according to root exposure height and mean sensitivity score. To ensure a homogeneous distribution of these two factors across study groups, teeth will be ordered by sensitivity scores and divided into two clusters: one with lower scores and the other with higher scores. These clusters will then be subdivided by smaller/larger exposure size, resulting in four groups: (a) lower sensitivity and smaller exposures, (b) lower sensitivity and larger exposures, (c) higher sensitivity and smaller exposures, and (d) higher sensitivity and larger exposures. This stratified randomization method was based on another parallel clinical trial.[31] Stratification will be performed approximately every 48 selected teeth for methodological feasibility.

In cases where experimental treatments do not reduce dentin hypersensitivity, patients will receive the product with the best performance to avoid further damage.

3.4 Clinical Procedures

After baseline assessments, a lip and cheek retractor will be placed. The tooth receiving the desensitizing agent will be isolated with cotton rolls and dried with an air jet, with moisture controlled by suction. The assigned product will be applied in two layers with a microbrush and left on the surface for 10 minutes. Light-cured products will be photoactivated for 10 s (LED Radii-cal, SDI Brasil Indústria e Comércio LTDA, São Paulo, Brazil). Afterward, cotton rolls and the retractor will be removed.

At the end of the study, if experimental treatments are ineffective in reducing dentin hypersensitivity, patients will be treated with the product that shows the best results to avoid further complications.

3.5 Assessments

DH intensity will be reassessed using the aforementioned scales at 7, 15, and 30 days, as well as 6 and 12 months post-treatment.[22,32]

3.6 Statistical Analyses

The results of DMFT, VPI, GBI indices, and root exposure height will be presented descriptively. Correlation between the sensitivity assessment methods used (CoVAS and VAS) will be analyzed using Spearman's correlation coefficient. Comparisons among treatments at the same evaluation time and among time points within the same treatment will be analyzed using equality tests for two proportions, with a significance level of 5%.

4. Implementation schedule

**Steps** / *Months*	**A**	**B**	**C**	**D**	**E**	**F**
1	**X**	**X**				
2	**X**	**X**				
3	**X**	**X**				
4	**X**	**X**				
5	**X**	**X**	**X**			
6	**X**		**X**	**X**		
7	**X**			**X**		
8	**X**			**X**		
9	**X**			**X**		
10	**X**			**X**		
11	**X**			**X**		
12	**X**			**X**	**X**	**X**
13	**X**				**X**	**X**
14	**X**					**X**
15	**X**					**X**
16	**X**					**X**

Step A – Bibliographic survey of all studies to search the literature on root exposure, dentin hypersensitivity, and desensitizing treatments used in prospective studies.
Step B – Patient selection; initial evaluation; product application; evaluation at 7, 15, and 30 days.
Step C – Statistics and data analysis up to 30 days
Step D – 6- and 12-month evaluation; tabulation of final data; statistical analyses.
Step E – Drafting of the final scientific report
Step F – Drafting of scientific articles.

5. References

1- Que, K., Guo, B., Jia, Z., Chen, Z., et al. A cross-sectional study: non-carious cervical lesions, cervical dentine hypersensitivity and related risk factors. J Oral Rehabil, 40:24-32, 2013.

2- Zeola, F.L., Soares, P.V., Cunha-Cruz, J. Prevalence of dentin hypersensitivity: systematic review and meta-analysis. J Dent, 81:1-6, 2019.

3- Felix, J., Ouanounou, A. Dentin hypersensitivity: Etiology, diagnosis, and management. Compend Contin Educ Dent, 40(10):653-657, 2019.

4- Chung, G., Jung, S.J., Oh, S.B. Cellular and molecular mechanisms of dental nociception. J Dent Res, 92:948-55, 2013.

5- Peumans, M., Politano, G., Van Meerbeek, B. Treatment of noncarious cervical lesions: When, why and how. Int J Esthet Dent, 15:16-42, 2020.

6- Yilmaz, H.G., Kurtulmus-Yilmaz, S., Cengiz, E. Long-term effect of diode laser irradiation compared to sodium fluoride varnish in the treatment of dentine hypersensitivity in periodontal maintenance patients: A randomized controlled clinical study. Photomed Laser Surg, 29: 721-5, 2011.

7- de Melo Alencar, C., de Franca Leite, K.L., Ortiz, M.I.G., Magno, M.B., et al. Morphological and chemical effects of in-office and at-home desensitising agents containing sodium fluoride on eroded root dentin. Arch Oral Biol, 110: 104619, 2019.

8- Kim, H.N., Kim, J.B., Jeong, S.H. Remineralization effects when using different methods to apply fluoride varnish in vitro. J Dent Sci, 13:360-366, 2018.

9- Pichaiaukrit, W., Thamrongananskul, N., Siralertmukul, K., Swasdison S. Fluoride varnish containing chitosan demonstrated sustained fluoride release. Dent Mater J, 38:1036-1042, 2019

10- Renno, A.C.M, Bossini, P.S.B, Crovace, M.C., Rodrigues, A.C.M, Zanotto, E.D., Parizotto, N.A. Characterization and in vivo biological performance of biosilicate. Biomed Res Int, 2013:141427, 2013.

11- Pintado-Palomino, K., Tirapelli, C. The effect of home-use and in-office bleaching treatments combined with experimental desensitizing agents on enamel and dentin. Eur J Dent, 9:66-73, 2015.

12- Tirapelli, C., Panzeri, H., Lara, E.H.G., Soares, R.G., Peitl, O., Zanotto, E.D. The effect of a novel crystallized bioactive glass-ceramic powder on dentine hypersensitivity: a long-term clinical study. J Oral Rehabil, 38(4):253-262, 2011

13- Tirapelli, C., Panzeri, H., Soares, G.R., Peitl, O., Zanotto, D.E. A novel bioactive glass-ceramic for treating dentin hypersensitivity. Braz Oral Res, 24(4):381-7, 2010.

14- Abbarin, N., Miguel, S.S., Holcroft, J., Iwasaki, K., Ganss, B. The enamel protein amelotin is a promoter of hydroxyapatite mineralization. J Bone Miner Res, 30(5):775–785, 2015.

15- Ikeda, Y., Neshatian, M., Holcroft, J., Ganss, B. The enamel protein ODAM promotes mineralization in a collagen matrix. Connect Tissue Res, 59(1):62-66, 2018.

16- Fouillen, A., Neves, J.S., Mary, C., Castonguay, J.D., Moffatt, P., Baron, C., Nanci, A. Interactions of AMTN, ODAM and SCPPPQ1 proteins of a specialized basal lamina that attaches epithelial cells to tooth mineral Sci Rep, 24(7):46683, 2017.

17- Patil, S.A., Naik, B.D., Suma, R. Evaluation of three different agents for in-office treatment of dentinal hypersensitivity: A controlled clinical study. Indian J Dent Res, 26(1):38-42, 2015.
18- Askari, M., Yazdani, R. Comparison of two desensitizing agents for decreasing dentin hypersensitivity following periodontal surgeries: A randomized clinical trial. Quintessence Int, 50(4):320-329, 2019.
19- Ravishankar, P., Viswanath, V., Archana, D., Keerthi, V., et al. The effect of three desensitizing agents on dentin hypersensitivity: A randomized, split-mouth clinical trial. Indian J Dent Res, 29:51-55, 2018.
20- Marto, C.M., Paula, A.B., Nunes, T., Pimenta, M., Abrantes, A.M., Pires, A.S., Laranjo, M., Coelho, A., Donato, H., Botelho, M.F., Ferreira, M.M., Carrilho, E. Evaluation of the efficacy of dentin hypersensitivity treatments-A systematic review and follow-up analysis. J Oral Rehabil, 46(10):952-990, 2019
21- Schulz, K.F., Altman, D.G., Moher, D. CONSORT 2010 statement: updated guidelines for reporting parallel group randomized trials. PLoS Med 7, e1000251, 2010.
22- Moura, G.F., Zeola, L.F., Silva, M.B., Sousa, S.C., Guedes, F.R., Soares, P.V. Four-session protocol effectiveness in reducing cervical dentin hypersensitivity: a 24-week randomized clinical trial. Photobiomodul Photomed Laser Surg, 37(2):177-123, 2019.
23- Ritter, A.V., Dias, W.L., Miguez, P., Caplan, D.J., Swift, E.J. Jr. Treating cervical dentin hypersensitivity with fluoride varnish: a randomized clinical study. J Am Dent Assoc, 137:1013-20, 2006.
24- Sgreccia P.C., Barbosa R.E.S., Damé-Teixeira N., Garcia F.C.P. Low-power laser and potassium oxalate gel in the treatment of cervical dentin hypersensitivity – a randomized clinical trial. Clin Oral Investig. https://10.1007/s00784-020-03311- 7 2020.
25- Fujimoto Y, Iwasa M, Murayama R, Miyazaki M, Nagafuji A, Nakatsuka T. Detection of ions released from S-PRG fillers and their modulation effect. Dent Mater J, 2010;29(4):392-397.
26- Peng, X., Xu, X., Li, Y., Cheng, L., Zhou, X., Ren, B. Transmission routes of 2019-nCoV and controls in dental practice. Int J Oral Sci, 12: https://10.1038/s41368-020-0075-9, 2020.
27- Kampf, G., Todt, D., Pfaender, S., Steinmann, E. Persistence of coronaviruses on inanimate surfaces and their inactivation with biocidal agents. J Hosp Infect, 104:246-251, 2020.
28- Rahal, V., Gallinari, M.O., Barbosa, J.S., Martins-Junior, R.L., dos Santos, P.H., Cintra, L.T.A., Briso, A.L.F. Influence of skin cold sensation threshold in the occurrence of dental sensitivity during dental bleaching: a placebo controlled clinical trial. J Appl Oral Sci, 26:e20170043, 2018.
29- Briso, A.L.F., Rahal, V., Azevedo, F.A., Gallinari, M.O., Gonçalves, R.S., Frascino, S.M.B, Santos, P.H.D., Cintra, L.T.A. Neurosensory analysis of tooth sensitivity during at-home dental bleaching: a randomized clinical trial. J Appl Oral Sci, 26:e20170284, 2018.
30- Shinohara, M.S., Carvalho, P.R.M., Neves Marcon, L., Gonçalves, D.F.M., Ramos, F.S.S., Fagundes, T.C. Randomized clinical trial of different adhesion strategies in noncarious cervical lesion restorations: 1-year follow-up. Quintessence Int, 51(5):352-

363, 2020.
31- Menezes-Silva, R., Velasco, S.R.M, Bastos,R.S, Molina, G., Honório, H.M., Frencken, J.E., Navarro, M.F.L. Randomized clinical trial of class II restoration in permanent teeth comparing ART with composite resin after 12 months. Clin Oral Investig, 23(9):3623-3635, 2019.
32- Sivaramakrishnan, G., Sridharan, K. Fluoride varnish versus glutaraldehyde for hypersensitive teeth: a randomized controlled trial, meta-analysis and trial sequential analysis. Clin Oral Investig, 23(1):209-220, 2019.
