# Supplementary material for: Longevity of different in-office treatments for dentin hypersensitivity: A 6-month randomized and parallel clinical trial
Source: PLoS One. 2026 Feb 17;21(2):e0342651. doi: 10.1371/journal.pone.0342651 (PMC12912554; doi:10.1371/journal.pone.0342651)
Supplement: S7 File — Clinical trial registration record in the Brazilian Registry of Clinical Trials (REBEC). (PDF) [file pone.0342651.s007.pdf]

BRASIL

ReBEC
Registro Brasileiro de Ensaios Clínicos

Estudo publicado

# RBR-5nw4zp Effect of different protocols in the treatment of tooth sensitivity

**Data de registro:** 11/05/2020 (dd/mm/yyyy)

**Última data de aprovação:** 14/05/2021 (dd/mm/yyyy)

## Tipo de estudo:

Intervenções

## Título científico:

**en**
Effect of different treatments on dentin hypersensitivity: evaluation of cytotoxicity and randomized clinical study

**pt-br**
Efeito de diferentes tratamentos na hipersensibilidade dentinária: avaliação da citotoxidade e estudo clínico randomizado

## Identificação do ensaio

- **Número do UTN:** U1111-1251-1091
- **Título público:**

  **en**
  Effect of different protocols in the treatment of tooth sensitivity

  **pt-br**
  Efeito de diferentes protocolos no tratamento da sensibilidade dentária

- **Acrônimo científico:**
- **Acrônimo público:**

  **en**
  PTDH

  **pt-br**
  PTHD

- **Identificadores secundários:**
  - **30122220.1.0000.5420**
    Orgão emissor: Plataforma Brasil
  - **3.988.387**
    Orgão emissor: Comitê de Ética em Pesquisa da Faculdade de Odontologia do Campus de Araçatuba - Universidade Estadual Paulista

## Patrocinadores

- **Patrocinador primário:** Universidade Estadual Paulista - FOA
- **Patrocinador secundário:**
  - **Instituição:** Universidade Estadual Paulista - FOA
- **Fontes de apoio financeiro ou material:**
  - **Instituição:** Universidade Estadual Paulista - FOA

## Condições de saúde

- **Condições de Saúde:**

| en | pt-br |
|---|---|
| Dentin Erosion; Dentin Sensitivity | Erosão dentinária; Sensibilidade Dentinária |

- **Descritores gerais para condições de saúde:**

| en | pt-br | es |
|---|---|---|
| **C07** Stomatognathic diseases | **C07** Doenças estomatognáticas | **C07** Enfermedades estomatognáticas |
| **C07** Stomatognathic diseases | **C07** Doenças estomatognáticas | **C07** Enfermedades estomatognáticas |

- **Descritores específicos para condições de saúde:**

| en | pt-br | es |
|---|---|---|
| **SP4.036.387.203** Erosion | **SP4.036.387.203** Erosão | **SP4.036.387.203** Erosión |
| **C07.793.266** Dentin Sensitivity | **C07.793.266** Sensibilidade da Dentina | **C07.793.266** Sensibilidad de la Dentina |

## Intervenções

- **Intervenções:**

| en | pt-br |
|---|---|
| One hundred and ninety-two teeth (48 for each treatment) with exposed root with dentin hypersensitivity not cavitated or beginning to cavitate (up to 1mm deep, without the need for restoration) will be treated in a randomized and parallel study. Each patient will be treated with the same desensitizing protocol. The degree of dentin sensitivity will be analyzed using the visual analogue scale (VAS), and computerized visual scale (CoVAS), at baseline (prior to treatment), and at 7, 15, 30 days, 6 and 12 months after treatment. | Cento e noventa e dois dentes (48 por tratamento) com exposições radiculares não cavitadas ou com início de cavitação (até 1mm de profundidade, sem necessidade de restauração) serão tratados por meio de um estudo paralelo randomizado. Cada paciente será tratado com o mesmo protocolo dessensibilizante. O grau de sensibilidade dentinária será analisado por meio da escala visual analógica (VAS) e escala visual computadorizada (CoVAS), antes do tratamento (baseline) e 7, 15, 30 dias, 6 e 12 meses após o tratamento. |

- **Descritores para as intervenções:**

| en | pt-br | es |
|---|---|---|
| **D27.505.696.663.850.014.640** Dentin Desensitizing Agents | **D27.505.696.663.850.014.640** Dessensibilizantes Dentinários | **D27.505.696.663.850.014.640** Desensibilizantes Dentinarios |

## Recrutamento

- **Situação de recrutamento:** Recrutando
- **Países de recrutamento**
  - Brasil
- **Data prevista do primeiro recrutamento:** 05/05/2021 (dd/mm/yyyy)
- **Tamanho da amostra alvo:** **Gênero para inclusão:** **Idade mínima para inclusão:** **Idade máxima para inclusão:**

| | | | |
|---|---|---|---|
| 51 | - | 20 Y | 70 Y |

- **Critérios de inclusão:**

**en**
For the in vivo study, patients will be selected who:Age between 20 and 70 years old; regardless of sex; Good health; with no history of allergies to dental products; Presence of at least 1 non-cavitated root exposures or with the beginning of cavitation (without need for restoration), which is sensitive to the jet from a distance of 10 cm.

**pt-br**
Para o estudo in vivo, serão selecionados pacientes que:Idade entre 20 e 70 anos; independente do sexo; Boa saúde, sem história de alergias a produtos odontológicos; Presença de no mínimo 1 exposição radiculares não cavitadas ou com início de cavitação (sem necessidade de restauração), que apresentem sensibilidade ao jato de ar em uma distância de 10cm.

- **Critérios de exclusão:**

**en**
For in vivo study:Pregnant, lactating or smokers; Presence of active caries lesions; Use of desensitizing agents in the last 6 months; Active and untreated periodontal disease; Use of orthodontic appliance or removable partial denture with clamp on the tooth to be evaluated;Severe bruxism, with more than 50% wear

**pt-br**
Para o estudo in vivo: Gestantes, lactantes ou fumantes; Presença de lesões de cárie ativas; Uso de agentes dessensibilizantes nos últimos 6 meses; Doença periodontal ativa e sem tratamento; Uso de aparelho ortodôntico ou prótese parcial removível com grampo no dente a ser avaliado;Bruxismo severo, com mais de 50% de desgaste

## Tipo de estudo

- **Desenho de estudo:**

| Programa de acesso expandido | Enfoque do estudo | Desenho da intervenção | Número de braços | Tipo de mascaramento | Tipo de alocação | Fase do estudo |
|---|---|---|---|---|---|---|
| | Tratamento | Paralelo | 4 | Unicego | Randomizado controlado | N/A |

## Desfechos

- **Desfechos primários:**

**en**
Expect to find an innovative and effective clinical protocol,verified through tooth sensitivity and analysis, for treatment to dentin hypersensitivity.

**pt-br**
Espera-se encontrar protocolo clínico inovador e eficaz, verificado por meio da análise da sensibilidade dentária, um tratamento eficaz e duradouro para hipersensibilidade dentinária.

- **Desfechos secundários:**

**en**
Secondary outcomes are not expected

**pt-br**
Não são esperados desfechos secundários

## Contatos

- **Contatos para questões públicas**
  - **Nome completo:** Fernanda de Souza e Silva Ramos
  - 
    - **Endereço:** Rua José Bonifácio, 1193
    - **Cidade:** Araçatuba / Brazil
    - **CEP:** 16015-050
  - **Fone:** +55-018-36363253
  - **Email:** fer_ramos_fer@hotmail.com
  - **Afiliação:** Universidade Estadual Paulista - FOA
  - **Nome completo:** Ticiane Cestari Fagundes
  - 
    - **Endereço:** Rua José Bonifácio, 1193
    - **Cidade:** Araçatuba / Brazil
    - **CEP:** 16015-050
  - **Fone:** +55-018-36363347
  - **Email:** ticiane.fagundes@unesp.br
  - **Afiliação:** Universidade Estadual Paulista - FOA
- **Contatos para questões científicas**
  - **Nome completo:** Fernanda de Souza e Silva Ramos
  - 
    - **Endereço:** Rua José Bonifácio, 1193
    - **Cidade:** Araçatuba / Brazil
    - **CEP:** 16015-050
  - **Fone:** +55-018-36363253
  - **Email:** fer_ramos_fer@hotmail.com
  - **Afiliação:** Universidade Estadual Paulista - FOA
  - **Nome completo:** Ticiane Cestari Fagundes
  - 
    - **Endereço:** Rua José Bonifácio, 1193
    - **Cidade:** Araçatuba / Brazil
    - **CEP:** 16015-050
  - **Fone:** +55-018-36363347
  - **Email:** ticiane.fagundes@unesp.br
  - **Afiliação:** Universidade Estadual Paulista - FOA
- **Contatos para informação sobre os centros de pesquisa**
  - **Nome completo:** Fernanda de Souza e Silva Ramos
  - 
    - **Endereço:** Rua José Bonifácio, 1193
    - **Cidade:** Araçatuba / Brazil
    - **CEP:** 16015-050
  - **Fone:** +55-018-36363253
  - **Email:** fer_ramos_fer@hotmail.com
  - **Afiliação:** Universidade Estadual Paulista - FOA
  - **Nome completo:** Ticiane Cestari Fagundes
  - 
    - **Endereço:** Rua José Bonifácio, 1193
    - **Cidade:** Araçatuba / Brazil
    - **CEP:** 16015-050
  - **Fone:** +55-018-36363347
  - **Email:** ticiane.fagundes@unesp.br
  - **Afiliação:** Universidade Estadual Paulista - FOA

## Links adicionais:

- Revisão anterior
- Download no formato ICTRP

Total de Ensaios Clínicos 16871.

Existem 8298 ensaios clínicos registrados.

Existem 4671 ensaios clínicos recrutando.

Existem 276 ensaios clínicos em análise.

Existem 5739 ensaios clínicos em rascunho.

cadastre um novo usuário

notícias

sobre

ajuda

contato

equipe

links úteis

glossário

Organização Pan-Americana da Saúde World Health Organization FIOCRUZ SUS MINISTÉRIO DA SAÚDE GOVERNO FEDERAL BRASIL UNIÃO E RECONSTRUÇÃO
